# Nitrogen uptake, grain yield, and oil concentration of dwarf castor beans under nitrogen rates and inoculation of rhizobacteria in grasses–legumes rotation

**DOI:** 10.3389/fmicb.2024.1451514

**Published:** 2024-08-26

**Authors:** Isabela Martins Bueno Gato, Carlos Eduardo da Silva Oliveira, Arshad Jalal, Vitória de Almeida Moreira, Amr H. Hashem, Bruno Horschut de Lima, Gabriel da Silva Leite, Abdulaziz A. Al-Askar, Leandro Alves Freitas, Hamada AbdElgawad, Selton Vinicius Domingos Ferreira, Leticia de Jesus Santana, Andréa de Castro Bastos, Fernando Shintate Galindo, Tiago Zoz, Marcelo Carvalho Minhoto Teixeira Filho

**Affiliations:** ^1^Department of Plant Protection, Rural Engineering and Soils, School of Engineering, São Paulo State University - UNESP-FEIS, Ilha Solteira, São Paulo, Brazil; ^2^University Unit of Cassilândia, Department of Agronomy, State University of Mato Grosso do Sul—UEMS, Cassilândia, Mato Grosso do Sul, Brazil; ^3^Botany and Microbiology Department, Faculty of Science, Al-Azhar University, Cairo, Egypt; ^4^Department of Botany and Microbiology, Faculty of Science, King Saud University, Riyadh, Saudi Arabia; ^5^Integrated Molecular Plant Physiology Research, Department of Biology, University of Antwerp, Antwerp, Belgium; ^6^Department of Crop Production, College of Agricultural and Technology Sciences, São Paulo State University (UNESP), Dracena, São Paulo, Brazil; ^7^University Unit of Mundo Novo, Department of Crop Science, State University of Mato Grosso do Sul—UEMS, Mundo Novo, Mato Grosso do Sul, Brazil

**Keywords:** *Ricinus communis* L., nitrogen fertilization, *Azospirillum brasilense*, *Bacillus subtilis*, *Pseudomonas fluorescens*, oil yield

## Abstract

**Introduction:**

Plant growth-promoting bacteria (PGPB) have been primarily studied for atmospheric nitrogen (N) fixation but they also have the capacity to improve nutrition and yield of crop plants.

**Methods:**

Therefore, the objective of this research was to investigate the effects of inoculation with PGPB in association with different N rates on N uptake, grain yield, and oil concentration of dwarf castor beans in succession to legumes and grasses in Ilha Solteira, Brazil. The treatments consisted of N rates (0 to 180 kg ha^−1^ of N) and inoculation with three plant growth-promoting bacteria (*Azospirillum brasiliense, Bacillus subtilis*, and *Pseudomonas fluorescens*, applied by leaf) and a control with no-inoculation.

**Results:**

The grain and oil yields of castor beans were increased by 20 and 40% at a rate of 103 kg ha^−1^ of N in succession to grasses as compared to without N application. In addition, the grain yield of castor bean after legumes was increased by 28, 64, and 40% with estimated rates of 97, 113, and 92 kg ha^−1^ of N in combination with inoculations of *A. brasilense*, *B. subtilis*, and *P. fluorescens* as compared to without N application, respectively. Shoot, grain, and total N uptake were improved with foliar inoculation of *A. brasilense*, *B. subtilis*, and *P. fluorescens* at the N rates of 45, 90, and 135 kg ha^−1^, respectively.

**Discussion and conclusions:**

Topdressing of N at the rate of 103 kg ha^−1^ and foliar inoculation in succession to grasses and 180 kg ha^−1^ of N without the effect of foliar inoculation in succession to legumes are recommended for higher grain and oil yield of castor beans. Foliar inoculations with *A. brasilense*, *B. subtilis*, and *P. fluorescens* increased grain yield under reduced use of N fertilizer by 44, 37, and 49% in dwarf castor cultivation in succession to legumes, potentially contributing to sustainable agriculture.

## 1 Introduction

Castor beans (*Ricinus communis* L.) is a member of the Euphorbiaceae family and is widely grown in many tropical countries. Castor bean is an important non-edible oilseed plant that contains approximately 40–60% oil and 80–90% ricinoleic acid, which is most commonly used for medicinal and industrial purposes (Mckeon, 2016; Das et al., 2023). It has a unique composition of fatty acids that can be used as one of the competitive feedstocks to produce biodiesel and high-value biopolymers (Ramanjaneyulu et al., 2017). Castor grows at an amazingly fast rate due to its easy adaptability to unfavorable conditions that appeal to its expansion to tropical conditions, being the major cultivated regions in India, China, and Brazil (Omotehinse and Omidih, 2021). Some of the agroclimatic zones of Brazil’s central and Cerrado regions are considered ideal conditions with the production of approximately 9.3 thousand metric tons of castor from an area of 5 thousand hectares (CONAB, 2024). The relative social and economic importance of castor in various industries demands the expansion of its cultivation under integrated and sustainable management of nitrogen (N) fertilizers and plant growth-promoting bacteria (PGPB).

Nitrogen is often one of the most limiting factors in the growth and development of crop plants (Galindo et al., 2024). Its deficiency affects the formation of photosynthesis and secondary metabolites, limiting light absorption and consequently plant growth (Urban et al., 2021). However, excessive or inadequate application of N fertilizers is contributing to the volatilization of the environment or leaching, which may pollute the atmosphere and water resources (Galindo et al., 2020). The inflation in socioeconomic costs of N fertilization has increased interest in the development of technologies such as inoculation with PGPB to reduce mineral fertilizer application (Lozada et al., 2018). The use of PGPB of the genera, *Azospirillum, Bacillus,* and *Pseudomonas* allows for crops with high-quality production, aiming at the progress of more sustainable technologies for agriculture under less impactful management (Mortinho et al., 2022). These inoculants exploit soil water and nutrients to increase root architecture and regulate different physiological processes that can contribute to higher use efficiency of nutrients under reduced fertilizer application (Moretti et al., 2020; Jalal et al., 2023a,b).

Castor beans can change the physical and chemical characterizations of soil such as decompression, soil structuring, and recycling nutrients from subsoil to surface soil, and consequently enhancing resource availability for the subsequent crop (Ferreira et al., 2006). The predecessor crop after legume is allowed with higher soil N availability due to higher biological nitrogen fixation (BNF) and straw decomposition (Silva et al., 2020; Te et al., 2022). In contrast, the predecessor crops after grasses/cereals may require greater N supply due to greater affinity for N, low BNF rate, and a higher C/N ratio in straw (Du et al., 2019; Galindo et al., 2020).

In the view of the abovementioned comprehensive literature, few studies have addressed the environmental and economic sustainability of castor cultivation, biomass residue utilization (Parascanu et al., 2019), and biodiesel production (Liang et al., 2013), but lack the impact of integrated use of N rates and foliar inoculation with PGPB on castor cultivation, nutrition, and yield in succussion to grasses/cereals and legumes. In this context, determining N rates that provide higher grain yield and oil yield of dwarf castor in a successor crop to grasses and legumes. In addition, the use of plant growth-promoting bacteria can manage the topdressing of N in dwarf castor beans. The objective of this research was to investigate the reduced rates of N in association with inoculation of *A. brasilense, B. subtilis,* and *P. fluorescens* for improving productive efficiency and nutrient uptake in succession to grasses and legumes in tropical conditions.

## 2 Materials and methods

### 2.1 Location and characterization of the experimental area

The research was carried out at the Research Station of the São Paulo State University (UNESP), Ilha Solteira, located in Selvíria–Mato Grosso do Sul–Brazil. The approximate geographical coordinates of the site were 51° 22’ W, 20° 22’ S, and an altitude of 335 m. This specific region is called “Brazilian Cerrado,” which consists of gramineous woody savanna.

The soil of the experimental site is classified as red dystrophic (Santos et al., 2018). The current experiment was conducted in a no-tillage system with successive leguminous crops (soybean/common beans, before castor bean was soybean) and grasses crops (corn/wheat, before castor bean was corn).

Soil chemical attributes in the 0.0–0.20 m layer were determined before the initiation of the castor beans experiment (van Raij et al., 2001). The following results were obtained: (a) in the area of succession to grasses: 31.4 mg dm^−3^ of P (resin); 4 mg dm^−3^ of S-SO_4_; 22 g dm^−3^ of OM; 5.7 of pH (CaCl_2_); K, Ca, Mg, and H + Al = 2.2, 22.0, 14.0, and 28.0 mmol_c_ dm^−3^, respectively; Cu, Fe, Mn, and Zn (DTPA) = 3.1, 27.0, 41.2, and 2.3 mg dm^−3^, respectively; 0.28 mg dm^−3^ of B (hot water); CEC = 66.2 mmol_c_ dm^−3^ and 58% of base saturation; (b) in succession to legumes: 37.0 mg dm^−3^ of P (resin); 3 mg dm^−3^ of S-SO_4_; 25 g dm^−3^ of OM; 5.7 of pH (CaCl_2_); K, Ca, Mg, and H + Al = 4.4, 37.0, 24.0, and 31.0 mmol_c_ dm^−3^, respectively; Cu, Fe, Mn, and Zn (DTPA) = 5.5, 24.0, 84.7, and 2.5 mg dm^−3^, respectively; 0.32 mg dm^−3^ of B (hot water); CEC = 96.4 mmol_c_ dm^−3^ and 68% of base saturation.

**Figure 1:**
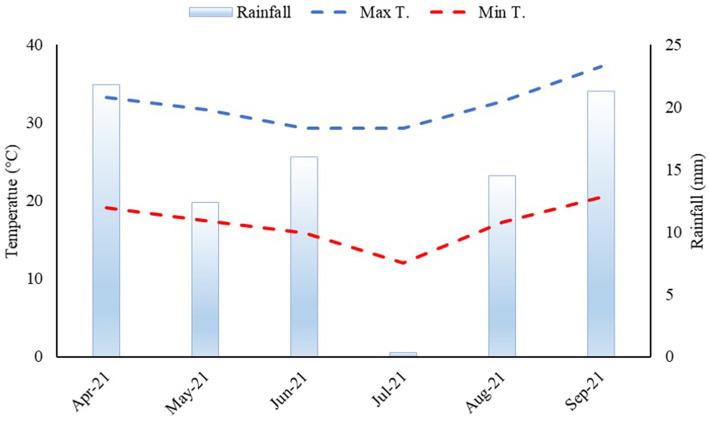
Rainfall, maximum, and minimum temperatures were acquired from the weather station of the Extension Research Farm of the Faculty of Engineering—UNESP during a dwarf castor cultivation period from April to September 2021.

The climate region is Aw as per the Köppen classification, which has been characterized as humid tropical with a rainy season in summer and a dry season in winter. The mean annual rainfall is 1,370 mm, the mean annual temperature is 23.5°C, and the relative humidity is between 70 and 80%. Mean rainfall and maximum and minimum temperatures during the cultivation of dwarf castor beans were monitored, as shown in Figure 1.

### 2.2 Experimental design and treatments

The experiment was designed in randomized blocks, arranged in a 5×4 factorial scheme with four replications. The treatments consisted of five N rates (0, 45, 90, 135, and 180 kg ha^−1^ of N, applied from urea as a source) and inoculation with three plant growth-promoting bacteria (*Azospirillum brasiliense, Bacillus subtilis,* and *Pseudomonas fluorescens*, applied by leaf) and a control with no-inoculation. The experimental field has a history with successive cultivation of legumes in the last 7 harvests (soybean/common bean—2017/2018, soybean/common bean—2018/2019, soybean/common bean—2019/2020, and soybean—2020/2021) and with successive cultivation of grasses in the last 7 harvests (corn/wheat—2017/2018, corn/wheat—2018/2019, corn/wheat—2019/2020, and corn—2020/2021). This allows us to determine and optimize the best rate of N in association with foliar inoculation of PGPB in dwarf castor beans.

### 2.3 Installation and management of the experiment

The castor bean seeds were mechanically sown in April 2021 with rows space of 0.90 m. The plots consisted of eight rows of 5.0 m length with plants spaced 0.30 m apart. The useful area consisted of four central lines, disregarding 0.50 m at both ends of each line. In addition, the sowing seeds were chemically treated with Standk Top^®^ (fungicide and insecticide) at the rate of 2 g kg^−1^ of seeds an hour before the plantation.

The castor beans hybrid “MIA” from Kaiima^®^, São Paulo, Brazil with an average productivity of 1,400 kg ha^−1^ in a non-irrigated area and 3,000 kg ha^−1^ in an irrigated area was used in the experiment. It has a life cycle of 120–150 days, height of 100–140 cm, adapted to mechanized harvesting, seed oil content is approximately 45% ± 2%, and has a nematocidal effect, controlling nematodes of the *Meloidogyne incognita*, *M. javanica,* and *Pratylenchus brachyurus* species.

Weed management was carried out with pre-emergence herbicide Dual Gold^®^ at a rate of 1.5 L ha^−1^ and post-emergence herbicide Gladium^®^ at a rate of 20 g ha^−1^, as recommended for castor bean or soybean crops. The recommended mineral fertilization was carried out in the sowing furrows at the rate of 250 kg ha^−1^ of NPK (8–28-16), providing 20 kg ha^−1^ of N, 70 kg ha^−1^ of P_2_O_5,_ and 40 kg ha^−1^ of K_2_O. The topdressing of N fertilizer was performed as per treatments, using urea (46% N) as a fertilizer source.

The supply of water was carried out by a fixed conventional sprinkler irrigation system with an average sprinkler precipitation of 3.3 mm h^−1^ and an average irrigation depth of 13 mm according to the necessity of the crop.

### 2.4 Plant growth-promoting bacterial strains and their application

The PGPB treatments were applied as a foliar spray at the fourth-leaf vegetative growth stage (V4) of the dwarf castor beans. The solution was diluted with tap water and sprayed in the morning with the aid of a backpack sprayer at a flow rate of 300 L ha^−1^. The droplet size was carefully regulated to prevent rolling-off of the leaf surface. We did not find an optimized foliar dose–response of PGPB, hence, doses for the foliar spray of the inoculants were selected following the manufacturer recommendations^1^ and previous experiments with PGPB via seeds inoculation (Rosa et al., 2022; Jalal et al., 2023b).

The foliar application with *Azospirillum brasilense* strains, Ab-V5 and Ab-V6, was carried out at a rate of 100 mL of liquid inoculant ha^−1^ (which is equivalent to 100 mL of inoculant per 37,000 castor bean seeds, when applied to seeds) making a concentration of 2 × 10^8^ forming unit of colony (CFU) mL^−1^. Inoculation with *Bacillus subtilis* strain CCTB04 via leaf was performed at a rate of 100 mL of liquid inoculant ha^−1^ (equivalent to 100 mL of inoculant per 37,000 castor bean seeds, when applied by seeds) to get a concentration of 1 × 10^8^ CFU mL^−1^. Inoculation with *Pseudomonas fluorescens* strain CCTB03 via leaf was performed at a rate of 100 mL of liquid inoculant ha^−1^ (equivalent to 100 mL of inoculant per 37,000 castor bean seeds, when applied by seeds) to get a concentration of with 1 × 10^8^ CFU mL^−1^. All these inoculant strains are commercially registered with the Ministry of Livestock and Agriculture (MAPA), Brazil, and available under the trade names of AzoTotal^®^ (MAPA PR Registration: 93923–10,074-1; *A. brasilense*), Vult^®^ (MAPA PR Registration: 001593–8.000004; *B. subtilis*) and Audax^®^ (MAPA PR registration: 16920; *P. fluorescens*).

### 2.5 Data collection and sample processing

The number of primary, secondary, and total racemes per plant was quantified at the end of the castor beans cycle. The number of grains was quantified by counting grains in primary and secondary racemes. A hundred-grain weight was estimated from the mean weight of one hundred grains by collecting and weighing 100 grains from each plot. The hundred grains weight was collected in both seasons, succession to cultivation of grasses (100W-G) and succession to legumes (100W-L). The grain yield was quantified by harvesting two central lines, threshed, and processed to measure the grain weight of each treatment with a precise scale.

The oil content of castor bean seeds was determined through the oil capture method by cold pressing. A 100 g of shelled castor beans were weighed and placed in a filter press adapted with a 10-ton mechanical press. The concentration of oil was weighed with a semi-analytical balance (g). The oil content in triplicate is expressed in percentage (%) of the product, adopting the simple arithmetic mean as the result. Oil yield was calculated via the formula.


$$\mathrm{Oil yield}\;\left(\mathrm{kg}\;{\mathrm{ha}}^{-1}\right)=\mathrm{Oil production}\;\left(\mathrm{kg}\;{\mathrm{ha}}^{-1}\right)\times \mathrm{Oil content}\;\left(\%\right)$$

In addition, plant material was dried, weighed, and ground in a Wiley mill to determine N concentrations (Malavolta et al., 1997) in the shoot and grain of dwarf castor beans. The shoot N uptake and grain N uptake of the plants were calculated from the fraction of shoot dry matter and grain yield through the following equations:


$$\begin{array}{c} \mathrm{Shoot}\;N\;\mathrm{uptake}\;\left(\mathrm{kg}\;{\mathrm{ha}}^{-1}\right)=\mathrm{shoot}\;\mathrm{dry}\;\mathrm{matter}\left(\mathrm{kg}\;{\mathrm{ha}}^{-1}\right)\\ \times \;N\;\mathrm{concentration}\;\left(g\;{\mathrm{ha}}^{-1}\right) \end{array}$$

$$\begin{array}{c} \mathrm{Grain}\;N\;\mathrm{uptake}\;\left(\mathrm{kg}\;{\mathrm{ha}}^{-1}\right)=\mathrm{Grain yield}\;\left(\mathrm{kg}\;{\mathrm{ha}}^{-1}\right)\\ \times \;N\;\mathrm{concentration}\;\left(g\;{\mathrm{ha}}^{-1}\right) \end{array}$$

### 2.6 Statistical analysis

The results were analyzed using the F test in the analysis of variance. When the F test indicated significant differences, the means were compared using Tukey’s test with a significance level of *p* < 0.05. This comparison was performed for the means of inoculations with PGPB while adjusting regression equations for the effect of nitrogen rates. All the statistical analyses were performed using the Sisvar statistical program (Ferreira, 2019).

## 3 Results

### 3.1 Effect of N rates and inoculations on the productive components

**Figure 2:**
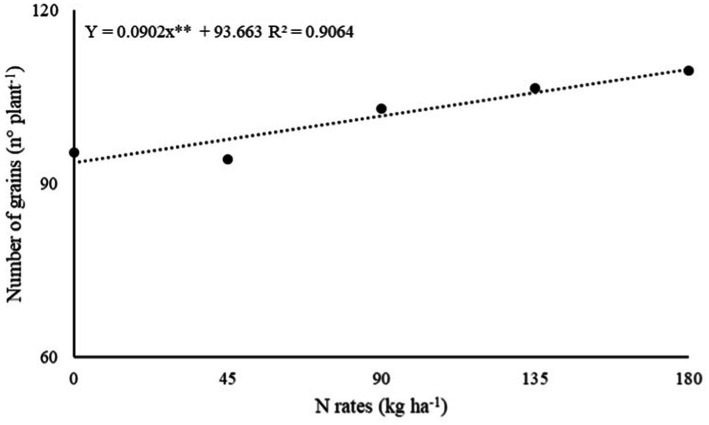
Effect of N rates under number of grains of dwarf castor grown in succession to grasses in a no-tillage system.

The effect of inoculations with PGPB and N rates and their interaction were not significant (*p* > 0.05) on the number of racemes (NR) and number of grains (NG) per plant of dwarf castor bean in succession to grasses and legumes (Table 1). The number of grains of castor beans in succession to grasses (NG-G) was adjusted to a linear increase in N rates (Table 1). The increasing N fertilizer in topdressing at a rate of 180 kg ha^−1^ was observed with higher calculated NG-G (109.6 grains) per plant of castor beans (Figure 2).

**Table 1:** Effect of nitrogen rates and inoculations of PGPB on the castor beans number of racemes (NR-G), number of grains (NG-G), and weight of 100 grains (100 W-G) in succession to grasses and number of racemes (NR-L), number of grains (NG-L), and weight of 100 grains (100 W-L) in succession to legumes.

Treatments	NR-G	NG-G	100 W-G	NG-L	NR-L	100 W-L
	N° plant^−1^		g	N° plant^−1^		g
Foliar inoculation						
Non-inoculation	5.83 a	97.70 a	38.36 a	82.41 a	5.40 a	36.50 ab
*A. brasilense*	6.08 a	103.27 a	36.61 a	93.17 a	5.13 a	35.75 b
*B. subtilis*	6.00 a	103.48 a	37.22 a	84.19 a	5.27 a	37.56 a
*P. fluorescens*	5.77 a	102.67 a	36.50 a	90.79 a	5.27 a	37.59 a
Nitrogen (N) rates (kg ha^−1^)						
0	5.87	95.44	36.45	81.22	5.17	36.33
45	6.15	94.29	37.30	89.59	5.21	36.79
90	5.94	103.04	37.39	83.78	5.44	36.37
135	6.10	106.50	36.80	97.37	5.50	37.24
180	5.54	109.63	37.93	86.24	5.02	37.51
Mean	5.92	101.78	37.17	87.64	5.26	36.85
Standard error (±)	0.21	4.93	1.11	4.48	0.18	1.15
Source of variance						
Block	0.03*	0.07ns	0.05*	0.62ns	0.00**	0.08ns
Inoculation (I)	0.86ns	0.75ns	0.06ns	0.16ns	0.89ns	0.01**
N rates (R)	0.71ns	0.11ns	0.46ns	0.09ns	0.69ns	0.35ns
I*R	0.32ns	0.07ns	0.19ns	0.09ns	0.21ns	0.06ns
Linear	0.50ns	0.01**	0.19ns	0.20ns	0.99ns	0.07ns
Quadratic	0.30ns	0.86ns	0.95ns	0.23ns	0.23ns	0.62ns
CV (%)	22.30	18.92	6.38	19.75	20.28	5.32

ns, not significant, * and ** significant at *p* < 0.05 and *p* < 0.01 probability using the *F* test. Distinct letters in the column differ from each other using Tukey’s test. PGPB, plant growth-promoting bacteria; CV, coefficient of variance.

In addition, the effect of inoculation with PGPB and N rates, and their interaction was not significant (*p* > 0.05) on 100 grains weight in succession to grasses (100W-G). The 100 grains weight in succession to legumes (100W-L) was significant (*p* < 0.01) in the treatments with inoculations PGPB while not significant with N rates (Table 1).

**Figure 3:**
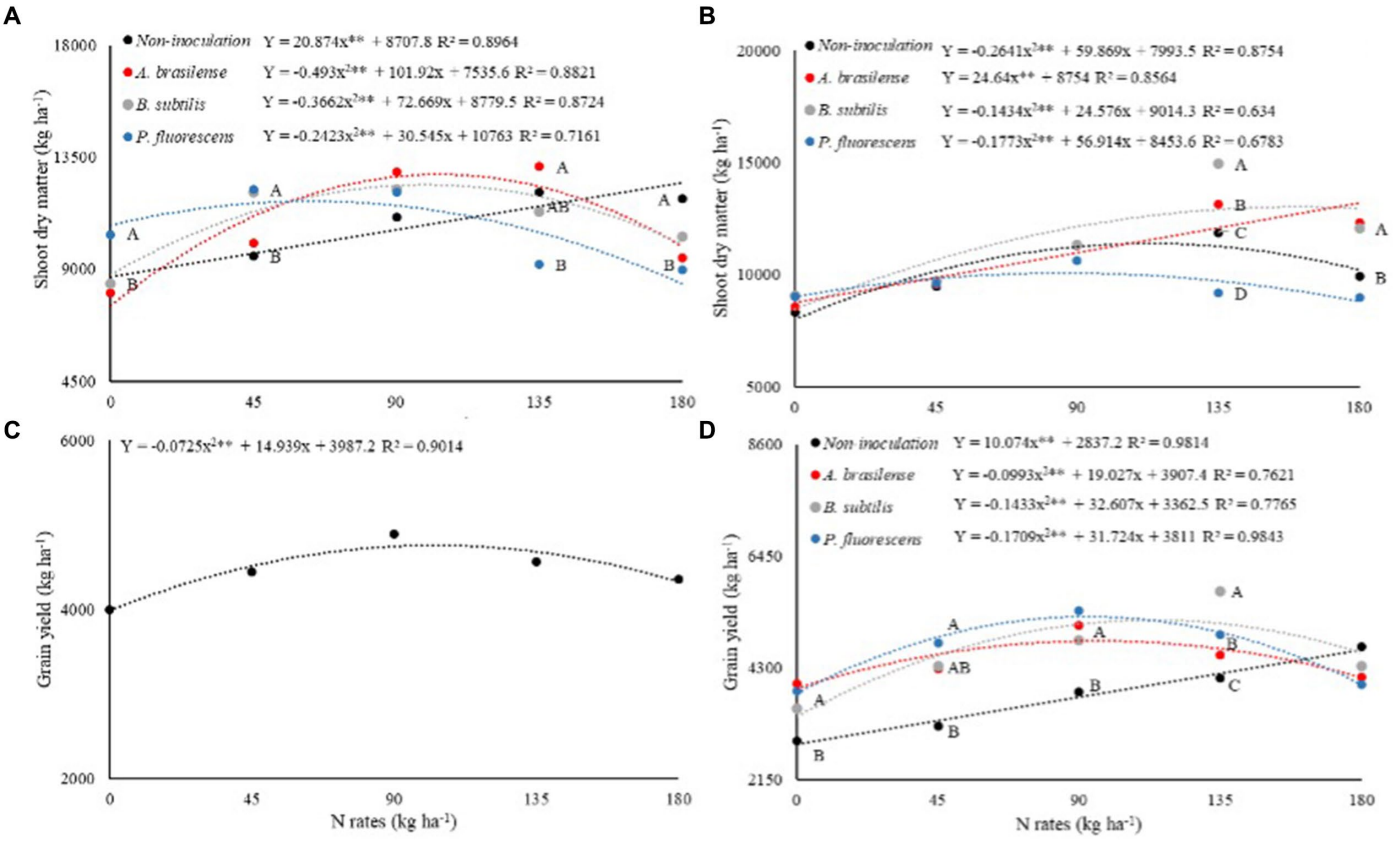
Effect of N rates and inoculations under shoot dry matter of dwarf castor plants grown in succession to grasses **(A)** and grown in succession to legumes **(B)**, grain yield of dwarf castor plants grown in succession to legumes **(D)**. Effect of N rates on grain yield of dwarf castor **(C)** cultivated in succession to cultivation with grasses in a no-tillage system.

Shoot dry mass of dwarf castor bean plants in succession to grasses (SDM-G) was significantly (*p* < 0.01) influenced by N rates and their interaction with inoculations of PGPB while shooting dry mass in succession to legumes (SDM-L) was significantly (*p* < 0.01) increased by interaction and treatments effects (Table 2). The SDM-G was increased with increasing N application in topdressing in the treatments without foliar inoculation. Inoculations with *A. brasilense, B. subtilis,* and *P. fluorescens* at estimated increasing N rates of 103, 99, and 63 kg ha^−1^ were observed with greater SDM-G as compared to N fertilization at a rate of 0 kg ha^−1^ (Figure 3A). The treatments with inoculation of *P. fluorescens* at the rates of 0 and 45 kg ha^−1^ of N increased SDM-G by 23 and 28% as compared to no-inoculation, while inoculation with *A. brasilense* in combination with 135 kg ha^−1^ of N increased SDM-G by 43% as compared to the treatments with inoculation of *P. fluorescens* (Figure 3A). The treatment with no foliar spray with PGPB was observed with greater SDM-G at the rate of 180 kg ha^−1^ of N as compared to the other treatments (Figure 3A).

**Table 2:** Effect of nitrogen rates and inoculations of PGPB on the castor beans shoot dry matter grown with grasses (SDM-G) and grain yield in succession to grasses (GY-G), shoot dry matter (SDM-L), and grain yield in succession to legume (GY-L).

Treatments	SDM-G	GY-G	SDM-L	GY-L
	cm	kg ha^−1^	cm	kg ha^−1^
Foliar inoculation				
Non-inoculation	10,586 a	3,339 d	10,122 b	3,743 b
*A. brasilense*	10,717 a	4,849 b	10,971 a	4,413 a
*B. subtilis*	10,870 a	5,397 a	11,421 a	4,556 a
*P. fluorescens*	10,567 a	4,214 c	9,483 c	4,590 a
Nitrogen (N) rates (kg ha^−1^)				
0	8,812	3,999	8,749	3,566
45	10,974	4,440	9,583	4,142
90	12,070	4,886	11,072	4,792
135	11,424	4,563	12,275	4,842
180	10,147	4,361	10,819	4,285
Mean	10,685	4,450	10,499	4,325
Standard error (±)	258.7	122.1	234.4	117.4
Source of variance				
Block	0.24ns	0.19ns	0.28ns	0.22ns
Inoculation (I)	0.58ns	0.00**	0.00**	0.00**
N rates (R)	0.00**	0.00**	0.00**	0.00**
I*R	0.00**	0.23 ns	0.00**	0.00**
Linear	0.00**	0.02*	0.00**	0.00**
Quadratic	0.00**	0.00**	0.00**	0.00**
CV (%)	7.27	9.92	8.06	5.21

ns, not significant, * and ** significant at *p* < 0.05 and *p* < 0.01 probability using the *F* test. Distinct letters in the column differ from each other using Tukey’s test. PGPB, plant growth-promoting bacteria; CV, coefficient of variance.

Shoot dry mass of dwarf castor beans in succession to legumes (SDM-L) was increased with inoculation with *A. brasilense* at increasing rates of N (Figure 3B). The treatments with no-inoculation, inoculation with *B. subtilis* and *P. fluorescens* increased SDM-L by 42, 65, and 18% at the estimated topdressing rates of 113, 85, and 160 kg ha^−1^ of N in relation to 0 kg ha^−1^ of N, respectively (Figure 3B). Inoculation with *B. subtilis* at a rate of 135 kg ha^−1^ of N was observed with 26% greater SDM-L as compared to without foliar spray of PGPB. Inoculation with *A. brasilense* and *B. subtilis* at a rate of 180 kg ha^−1^ of N increased SDM-L by 24 and 22% as compared to other inoculation and non-inoculation, respectively (Figure 3B).

The interaction for the grain yield of castor beans in succession to grasses (GY-G) was not significant whereas inoculation with PGPB and N rates and their interaction were observed significant (*p* < 0.01) for castor bean grain yield in succession to legumes (GY-L; Table 2). The GY-G was increased by 20% at the estimated topdressing rate of 103 kg ha^−1^ of N in relation to 0 kg ha^−1^ of N (Figure 3C). There was a linear increase in dwarf castor GY-L with increasing topdressing rates of N as compared to non-inoculated treatment (Figure 3D). Inoculations with *A. brasilense, B. subtilis,* and *P. fluorescens* increased GY-L by 28, 64, and 40% at the estimated rates of 97, 113, and 92 kg ha^−1^ of N in relation to 0 kg ha^−1^ of N, respectively (Figure 3D).

Inoculations with *A. brasilense, B. subtilis,* and *P. fluorescens* increased GY-L of castor beans by 37, 21, and 33%, while the same inoculations at the rate of 90 kg ha^−1^ of N increased GY-L by 33, 25, and 40% in relation to no-inoculation at 0 kg of N, respectively. Inoculation with *B. subtilis* increased GY-L by 41% at a rate of 135 kg ha^−1^ of N, whereas, inoculation with *P. fluorescens* at a rate of 45 kg ha^−1^ of N increased GY-L by 50% as compared to without foliar inoculation with PGPB (Figure 3D).

### 3.2 Effect of N rates and inoculations on the oil content and oil yield

**Figure 4:**
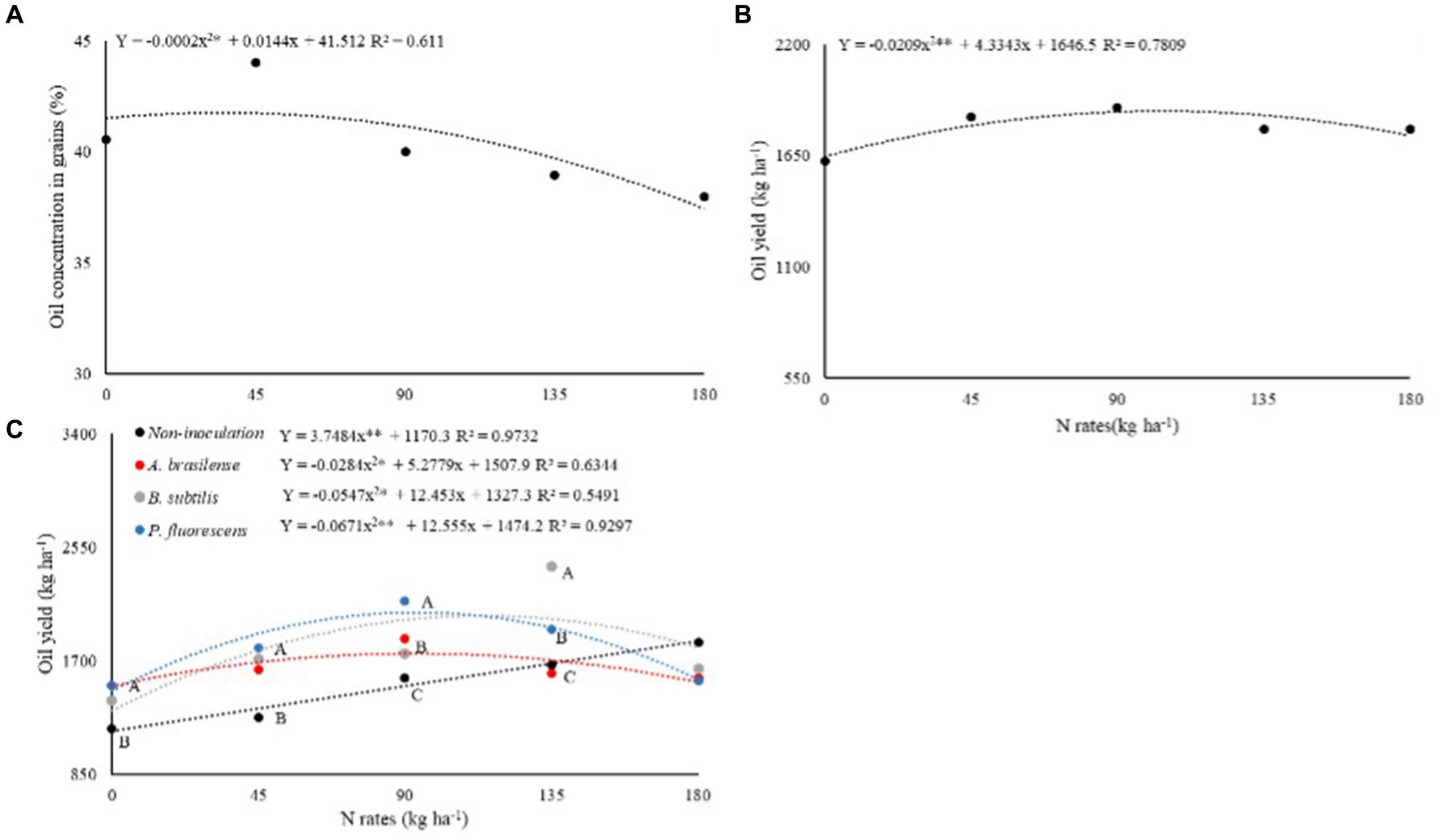
Effect of N rates under oil concentration in dwarf castor seeds **(A)** and on oil yield of dwarf castor **(B)** grown in succession to cultivation with grasses. Effect of N rates and inoculations on oil concentration in dwarf castor seeds grown in succession to legumes **(C)** in a no-tillage system.

The oil concentrations in castor bean grains in succession to grasses (%O-G) and legumes (%O-L) were significantly (*p* < 0.05) affected by N rates and inoculation with PGPB via leaf, respectively (Table 3). The %O-G was set to linear increase at the rate of 36 kg ha^−1^ of N and further increase in N rates led to the reduction of %O-G (Figure 4A).

**Table 3:** Effect of nitrogen rates and inoculations of PGPB on the castor beans seeds oil concentration (%O-G) and oil yield (OY-G) in succession to grasses and seeds oil concentration (%O-L) and oil yield (OY-L) in succession to legumes.

Treatments	%O-G	OY-G	%O-L	OY-L
	%	kg ha^−1^	%	kg ha^−1^
Foliar inoculation				
Non-inoculation	40.16 a	1,340 c	40.32 a	1,507 c
*A. brasilense*	40.64 a	1969 a	37.16 b	1,638 b
*B. subtilis*	39.48 a	2,127 a	39.06 ab	1783 a
*P. fluorescens*	40.26 a	1,693 b	38.90 ab	1788 a
Nitrogen (N) rates (kg ha^−1^)				
0	40.57	1,621	39.20	1,404
45	41.47	1842	39.03	1,605
90	38.70	1887	38.86	1833
135	38.97	1779	38.69	1906
180	40.96	1780	38.52	1,468
Mean	40.13	1782	38.86	1,679
Standard error (±)	2.15	93.26	1.89	80.14
Source of variance				
Block	0.95ns	0.32ns	0.10ns	0.09ns
Inoculation (I)	0.64ns	0.00**	0.02*	0.00**
N rates (R)	0.03*	0.01**	0.81 ns	0.00**
I*R	0.94ns	0.41ns	0.59ns	0.00**
Linear	0.45ns	0.13ns	0.49ns	0.00**
Quadratic	0.05*	0.00**	0.73 ns	0.00**
CV (%)	7.17	11.91	7.86	10.32

ns, not significant, * and ** significant at *p* < 0.05 and *p* < 0.01 probability using the *F* test. Distinct letters in the column differ from each other using Tukey’s test. PGPB, plant growth-promoting bacteria; SOV, source of variance; CV, coefficient of variance.

Castor oil yield in succession to grasses (OY-G) and legumes (OY-L) were significantly (*p* < 0.01) affected by N rates and inoculation with PGPB, while their interaction between two factors was significant (*p* < 0.01) for castor oil yields in succession to legumes (OY-L; Table 3). Higher OY-G was observed at the estimated rate of 103 kg ha^−1^ of N (Figure 4B). Inoculations with *A. brasilense, B. subtilis,* and *P. fluorescens* at the rate of 0 and 45 kg ha^−1^ of N increased OY-L by 27, 18, and 27%, and 28, 34, and 41% in relation to without foliar inoculation of PGPB, respectively. Inoculation with *P. fluorescens* at a rate of 90 kg ha^−1^ of N enhanced OY-L by 37% while inoculation with *B. subtilis* at a rate of 135 kg ha^−1^ of N improved OY-L by 44% as compared to no-inoculation (Figure 4C). Inoculations with *A. brasilense, B. subtilis,* and *P. fluorescens* increased OY-L by 23, 72, and 55% at the calculated topdressing rates of 92, 64, and 93 kg ha^−1^ of N in relation to 0 kg ha^−1^ of N, respectively (Figure 4C).

### 3.3 Effect of N rates and inoculations on the N concentration and N uptake

**Figure 5:**
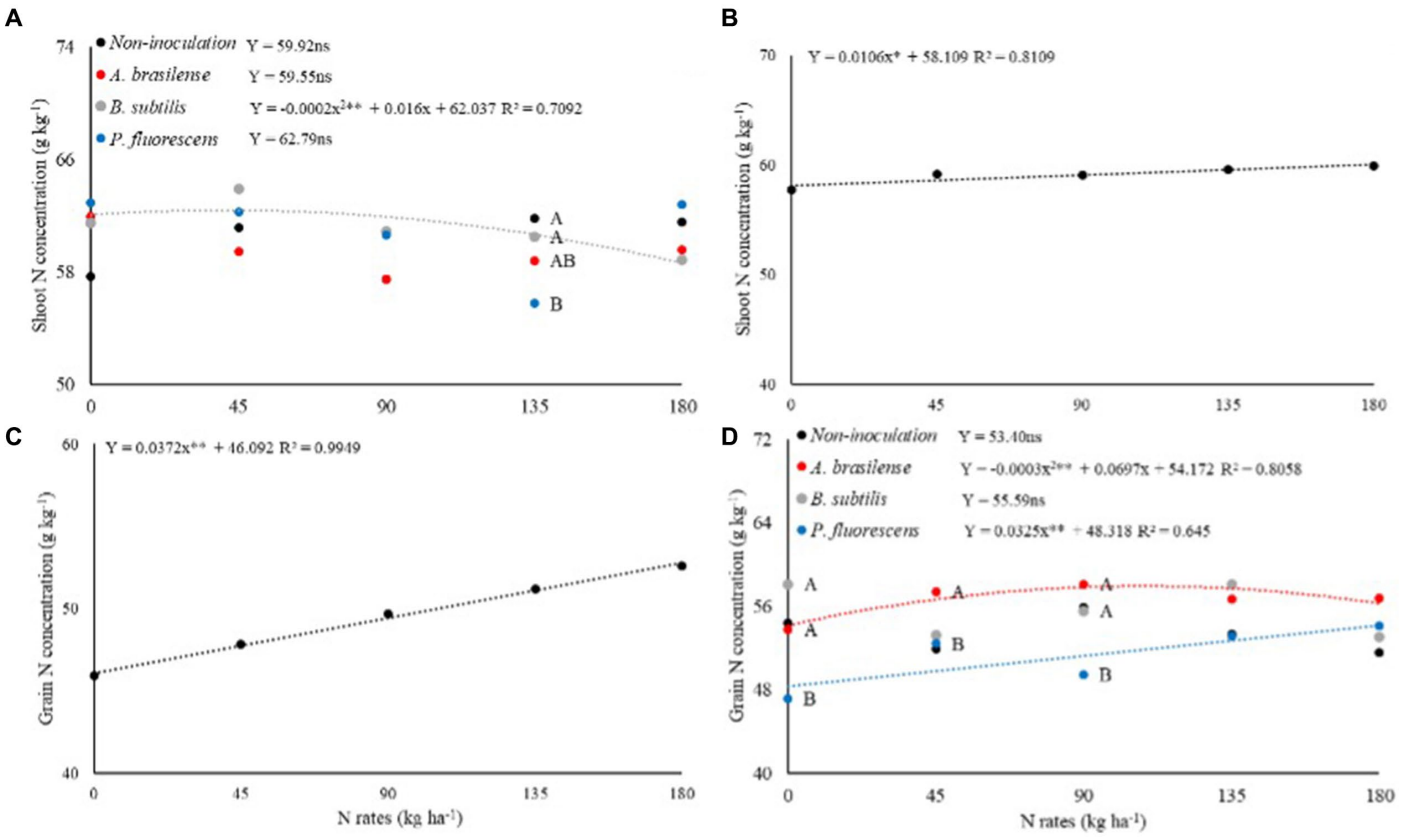
Effect of N rates and inoculations PGPB under shoot N concentration of dwarf castor plants grown in succession to grasses **(A)** and grain N concentration of dwarf castor grown in succession to legumes **(D)**. Effect of N rates under shoot N concentration of dwarf castor plants grown in succession to legumes **(B)** and grain N concentration of dwarf castor grown in succession to grasses **(C)** in a no-tillage system.

There was a significant effect (*p* < 0.05) of the interaction between N rates and PGPB inoculations on shoot N concentration of dwarf castor in succession to grasses (SNC-G; Table 4) while shooting N concentration in succession to legumes (SNC-L) set to linear regression (Figure 5A). The highest SNC-G of dwarf castor was observed with no-inoculation treatment and inoculation with *B. subtilis* at the N rate of 135 kg ha^−1^in relation to inoculation with *P. fluorescens*. The SNC-G of castor bean was increased to a maximum calculated N concentration of 62.36 g kg^−1^ up to a topdressing N rate of 40 kg ha^−1^ at inoculation with *B. subtilis* (Figure 5A). Higher SNC-L of dwarf castor was observed with increasing N supply in topdressing (Figure 5B).

**Table 4:** Effect of nitrogen rates and inoculations of PGPB on the castor beans shoot nitrogen concentration (SNC-G) and grain nitrogen concentration in succession to grasses (GNC-G), shoot nitrogen concentration (SNC-L), and grain nitrogen concentration in succession to legumes (GNC-L).

Treatments	SNC-G	GNC-G	SNC-L	GNC-L
	g kg^−1^			
Foliar inoculation				
Non-inoculation	59.92 a	48.39 a	60.23 a	53.24 b
*A. brasilense*	59.42 a	50.39 a	58.30 a	56.53 a
*B. subtilis*	60.85 a	50.75 a	59.21 a	55.59 a
*P. fluorescens*	61.11 a	48.21 a	58.51 a	51.24 c
Nitrogen (N) rates (kg ha^−1^)				
0	60.98	45.90	57.67	53.35
45	61.68	47.84	59.16	53.73
90	59.09	49.65	59.09	54.72
135	59.10	51.21	59.52	55.31
180	60.67	52.58	59.88	53.84
Mean	60.32	49.44	59.06	54.19
Standard error (±)	3.08	2.41	2.78	2.91
Source of variance				
Block	0.24ns	0.27ns	0.24ns	0.99ns
Inoculation (I)	0.29ns	0.07ns	0.06ns	0.00**
N rates (R)	0.09ns	0.00**	0.11ns	0.28ns
I*R	0.04*	0.26ns	0.22ns	0.00**
Linear	0.22ns	0.00**	0.01*	0.00**
Quadratic	0.15ns	0.69ns	0.44ns	0.00**
CV (%)	5.20	7.58	4.06	5.20

ns, not significant, * and ** significant at *p* < 0.05 and *p* < 0.01 probability using the *F* test. Distinct letters in the column differ from each other using Tukey’s test. PGPB, plant growth-promoting bacteria; CV, coefficient of variance.

The verified significant effect (*p* < 0.01) of N rates on grain nitrogen concentration in succession to grasses (GNC-G) and grain nitrogen concentration in succession to legumes (GNC-L) was significantly (*p* > 0.01) influenced by inoculation and interaction of N rates and inoculations (Table 4). The GNC-G of dwarf castor was increased with increasing N supply in topdressing (Figure 5C). Foliar inoculation with *A. brasilense, B. subtilis,* and without inoculation treatments at rates of 0 and 90 kg ha^−1^ of N were observed with higher GNC-L of dwarf castor bean than inoculation with *P. fluorescens*. Inoculation with *A. brasilense* at the topdressing N rate of 45 kg ha^−1^ was observed with greater castor bean GNC-L than other inoculations. The greater castor GNC-L was observed with increasing N supply in topdressing and foliar inoculation with *B. subtilis*. Inoculation with *A. brasilense* at an estimated topdressing N rate of 116 kg ha^−1^ was observed with maximum calculated GNC-L (58.22 g kg^−1^) in dwarf castor (Figure 5D).

There was a significant effect (*p* < 0.01) of N rates and interaction between N rates and inoculations on shoot N uptake dwarf castor plants in succession to grasses (SNU-G). There was a significant effect of N rates, inoculations with PGPB, and their interaction on shoot N uptake in succession to legumes (SNU-L) and grain N uptake in succession to grasses (GNU-G) and legumes (GNU-L) (Table 5).

**Table 5:** Effect of nitrogen rates and inoculations of PGPB on the castor bean shoot nitrogen uptake (SNU-G), grain nitrogen uptake (GNU-G) and total nitrogen uptake (TNU-G) in succession to grasses and shoot nitrogen uptake (SNU-L), grain nitrogen uptake (GNU-L), and total nitrogen uptake (TNU-L).

Treatments	SNU-G	GNU-G	TNU-G	GNU-L	SNU-L	TNU-L
	kg ha^−1^					
Foliar inoculation						
Non-inoculation	638.3 a	167.2 c	805.48 b	194.9 b	610.5 b	805.38 b
*A. brasilense*	625.2 a	248.7 a	873.82 a	220.2 a	640.9 ab	861.16 a
*B. subtilis*	643.4 a	273.4 a	916.82 a	228.6 a	676.7 a	905.40 a
*P. fluorescens*	618.4 a	208.5 b	826.87 ab	233.1 a	556.1 c	789.15 b
Nitrogen (N) rates (kg ha^−1^)						
0	505.8	195.2	701.05	176.9	504.9	681.79
45	649.2	216.5	865.64	207.3	566.9	774.29
90	712.6	248.5	961.08	240.9	654.1	894.99
135	681.1	232.8	913.86	253.6	732.4	986.04
180	607.9	229.2	837.12	217.3	646.9	864.27
Mean	631.3	224.4	855.75	219.21	621.1	840.28
Standard error (±)	26.24	14.18	38.89	19.56	24.44	36.22
Source of variance						
Block	0.47ns	0.11ns	0.48ns	0.85ns	0.59ns	0.60ns
Inoculation (I)	0.37ns	0.00**	0.00**	0.00**	0.00**	0.00**
N rates (R)	0.00**	0.00**	0.00**	0.00**	0.00**	0.00**
I*R	0.00**	0.04*	0.00**	0.00**	0.00**	0.00**
Linear	0.00**	0.00**	0.00**	0.00**	0.00**	0.00**
Quadratic	0.00**	0.00**	0.00**	0.00**	0.00**	0.00**
CV (%)	7.88	11.24	6.42	7.94	9.47	7.25

ns, not significant, * and ** significant at *p* < 0.05 and *p* < 0.01 probability using the *F* test. Distinct letters in the column differ from each other using Tukey’s test. PGPB, plant growth-promoting bacteria; CV, coefficient of variance.

**Figure 6:**
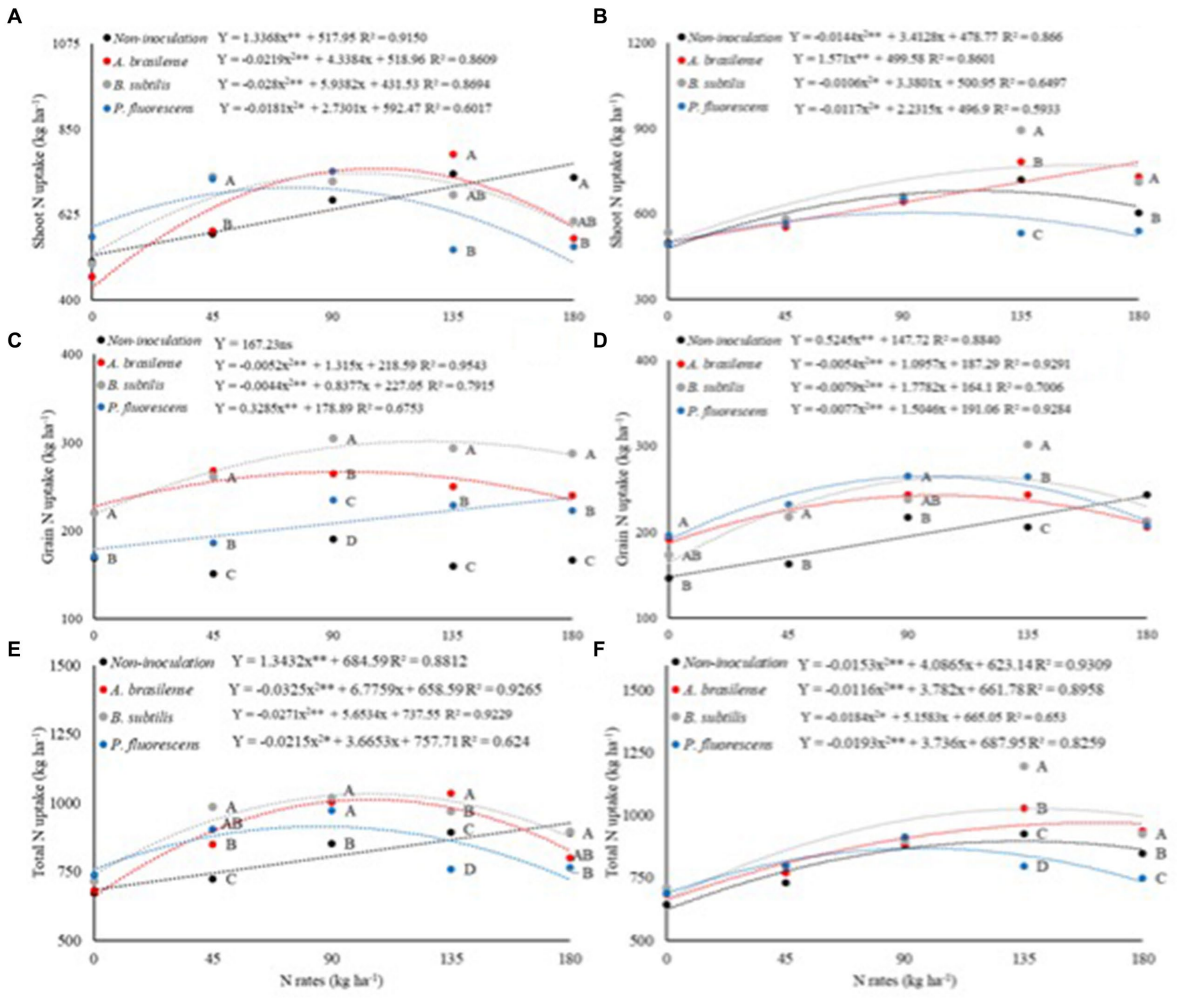
Effect of N rates and inoculations PGPB under shoot N uptake in dwarf castor plants grown in succession to grasses **(A)** and grown in succession to legumes **(B)**, grain N uptake of dwarf castor grown in succession to grasses **(C)** and grown in succession to legumes **(D)**, total N uptake of dwarf castor grown in succession to grasses **(E)**, and grown in succession to legumes **(F)** in a no-tillage system.

The SNU-G was increased with increasing N rates in topdressing in the no-inoculated treatments (Figure 6A). Inoculations with *A. brasilense, B. subtilis,* and *P. fluorescens* at the calculated rates of 99, 106, and 75 kg ha^−1^ of N improved SNU-G by 69, 44, and 30%, respectively. Inoculation with *B. subtilis* and *P. fluorescens* were with 26 and 25% higher SNU-G than no foliar inoculation. Inoculation with *A. brasilense* at a rate of 45 kg ha^−1^ of N and without foliar inoculation at a rate of 180 kg ha^−1^ of N were observed with higher SNU-G in relation to other inoculations. Inoculation with *A. brasilense* increased SNU-G by 47% in relation to inoculation with *P. fluorescens* at a rate of 135 kg ha^−1^ of N (Figure 6A).

Shoot N uptake in succession to legumes (SNU-L) was increased with increasing rates of N and inoculation with *A. brasilense* (Figure 6B). Treatments with no-inoculation and foliar inoculation with *B. subtilis* and *P. fluorescens* at the estimated topdressing rates of 118, 159, and 95 kg ha^−1^ of N increased SNU-L by 45, 67, and 32%, respectively (Figure 6B). Inoculation with *B. subtilis* at the rate of 135 kg ha^−1^ of N was observed with 24% SNU-L of dwarf castor as compared to non-inoculated. Inoculations with *A. brasilense* and *B. subtilis* at the rate of 180 kg ha^−1^ of N increased SNU-L by 21 and 18% as compared to no-inoculation, respectively (Figure 6B).

Grain N uptake in succession to grasses (GNU-G) was increased by 21 and 39% with inoculations of *A. brasilense* and *B. subtilis* at calculated rates of 95 and 126 kg ha^−1^ of N, respectively. There was a linear increase in GNU-G with increasing rates of N and inoculation with *P. fluorescens*. Inoculation with *B. subtilis* and *A. brasilense* at a rate of 45 kg ha^−1^ of N enhanced GNU-G by 39 and 60% as compared to no-inoculation. In addition, inoculation with *B. subtilis* at rates of 90, 135, and 180 kg ha^−1^ of N was observed with 60, 83, and 73% higher GNU-G than no-inoculation, respectively (Figure 6C).

There was an increase in the grain N uptake in succession to legumes (GNU-L) of dwarf castor as the N rates increased under the non-inoculated treatment. Inoculations of *A. brasilense, B. subtilis,* and *P. fluorescens* at the estimated rates of 101, 112, and 98 kg ha^−1^ of N increased GNU-L by 27, 37, and 35% in relation to 0 kg ha^−1^ of N, respectively (Figure 6D). Inoculation with *P. fluorescens* at a rate of 90 kg ha^−1^ of N increased GNU-L by 22% while inoculation with *B. subtilis* at a rate of 135 kg ha^−1^ of N increased GNU-L by 47% over non-inoculated treatments (Figure 6D).

There was an increase in the total N uptake in succession to grasses (TNU-G) with an increase in topdressing N rates in the non-inoculated treatments. Foliar inoculations with *A. brasilense, B. subtilis,* and *P. fluorescens*. The foliar inoculation of *A. brasilense, B. subtilis,* and *P. fluorescens* at the rate of 90 kg ha^−1^ of N provided higher TNU-G in relation to non-inoculated treatments. Inoculations with *A. brasilense* and *B. subtilis* at the rate of 135 kg ha^−1^ of N were observed with higher TNU-G than non-inoculated treatments (Figure 6E).

Foliar inoculation with *A. brasilense* and *B. subtilis* at the rates of 135 and 180 kg ha^−1^ of N was observed with higher total N uptake in succession to legumes (TNU-L) than non-inoculated (Figure 6F). The highest calculated TNU-L (896.01 kg ha^−1^) was verified at the estimated topdressing rate of 133 kg ha^−1^ of N under the non-inoculated treatments. Foliar inoculations with *A. brasilense, B. subtilis,* and *P. fluorescens* at estimated topdressing rates of 163, 140, and 96 kg ha^−1^ of N were observed with higher TNU-L (970.1, 1026.6, and 868.8 kg ha^−1^ of N), respectively (Figure 6F).

## 4 Discussion

Inoculation with *P. fluorescens, A. brasilense,* and *B. subtilis* is considered one of the diverse strategies of agricultural cultivation systems with a positive effect on shoot dry mass, grain yield, and other productive components (Andrade et al., 2019; Chu et al., 2020; Rosa et al., 2020; Akhtyamova et al., 2021; Jalal et al., 2021; Fonseca et al., 2022; Galindo et al., 2022; Jalal et al., 2023b), and a foliar inoculation with *A brasilense* in corn (Pereira et al., 2020). In addition, the use of these bacteria in association with N rates has been studied in several studies with the intention of reducing N consumption in the agriculture crop production system (Prasad and Babu, 2017; Galindo et al., 2020). However, there exists a research gap on the foliar inoculations with these bacteria and their ability to promote the growth of castor beans in Brazilian Cerrado.

The current results indicated that inoculation with *A. brasilense* at the rates of 90 and 135 kg ha^−1^ of N and inoculation with *P. fluorescens* at the rates of 0 and 45 kg ha^−1^ were observed with greater shoot dry matter of dwarf castor in succession to grasses. However, the highest shoot dry matter of dwarf castor beans in succession to legumes was noted with inoculations of *B. subtilis* and *A. brasilense* at the estimated rates of 135 and 180 kg ha^−1^ of N (Figures 3A,B). As previously reported, *A. brasilense, P. fluorescens,* and *B. subtilis* have the ability to produce different plant hormones in the root rhizosphere of crop plants that may promote the growth of roots and shoots (Mekonnen and Kibret, 2021).

The increased production of plant hormones by the inoculated plants could be linked to the higher distribution of photo-assimilates by the plant, especially in the period of greatest plant demand (flowering and fruiting), which may positively affect grain yield (Mortinho et al., 2022; Jalal et al., 2023a). This effect was observed in GY-G of castor bean with foliar inoculation of *B. subtilis* while inoculation with *A. brasilense*, *B. subtilis,* and *P. fluorescens* at the rates of 45 and 90 kg ha^−1^ of N were observed with higher GY-L of dwarf castor. The highest GY-L of dwarf castor was observed with inoculation of *B. subtilis* at the rate of 135 kg ha^−1^ of N (Figure 3). In addition, the highest OY-L of castor beans observed was with inoculation of *P. fluorescens* at a rate of 90 kg ha^−1^ of N and inoculation with *B. subtilis* at a rate of 135 kg ha^−1^ of N as compared to non-inoculated treatments (Figure 4).

The beneficial effects of inoculation with PGPB can be impaired by the high supply of N, which was the case in the present study that the highest rates of N impair the performance of PGPB in castor bean cultivation, by reducing grain yield and oil yields (Figure 4). The high supply of N fertilizer can increase the competition of bacteria and roots for the large amount of N present in the soil, which may affect the desired mechanisms of biological fixation of N_2_ by bacteria (Galindo et al., 2021).

An adequate supply of N can increase nutrient uptake by plants, as reported that PGPB can improve plant’s N acquisition through biological N fixation (BNF) (Fukami et al., 2018; Galindo et al., 2021) and greater root biomass through physiological changes in plants (Eckshtain-Levi et al., 2020). This could influence the ability of plant roots to penetrate the soil for greater water and nutrient absorption (Li et al., 2016). The present result verified greater N uptake with higher N supply in topdressing. The highest SNU-G was observed with inoculations of *B. subtilis* and *P. fluorescens* at a rate of 45 kg ha^−1^ of N and inoculation with *A. brasilense* at a rate of 135 kg ha^−1^ of N as compared to non-inoculated treatments (Figures 6A,B). Inoculation with *A. brasilense* and *B. subtilis* provided greater GNU-G at all rates of N; however, inoculation with *B. subtilis* and *P. fluorescens* was observed with greater GNU-L at rates of 45 and 135 kg ha^−1^ of N (Figures 6C,D). Although BNF is a determining factor for increasing N use efficiency and N uptake by plants, these bacteria are still functionally contributing to some other mechanisms (production of gibberellins, auxins, and cytokinins) that could increase plant growth and productivity (Fukami et al., 2018; Poveda and González-Andrés, 2021). Previous studies reported that inoculation of *A. brasilense* and *B. subtilis* has increased N use efficiency and recovery of applied N in cereal crops that contribute to sustainable grain production under reduced N fertilization (Galindo et al., 2022; Gaspareto et al., 2023).

The isolated inoculation of *A. brasilense* and *B. subtilis* proved to be effective in increasing the recovery of applied N, N use efficiency, shoot N uptake, grain N uptake, productive components, and the grain yield of wheat (Gaspareto et al., 2023). Curiously, it is possible to highlight the highest TNU-G of 51, 42, and 32% at rates of 104, 104, and 85 kg ha^−1^ of N under foliar inoculations of *A. brasilense, B. subtilis,* and *P. fluorescens,* respectively. There was an increase of 50, 68, and 33% in TNU-L under the inoculations of *A. brasilense, B. subtilis,* and *P. fluorescens* at rates of 163, 140, and 96 kg ha^−1^ of N in dwarf castor bean (Figures 6E,F). Castor bean plants are tolerant to water scarcity, which allows them to be cultivated in both arid regions and regions with adequate water availability. However, under the appropriate conditions of cultivation system and water supply, these plants exhibit enhanced development (Zoz et al., 2021). In addition, castor bean plants are responsive to nitrogen fertilization, with greater N acquisition by plants and being translocated to plant tissues (Cavalcante et al., 2020). Inoculations with *B. subtilis, P. fluorescens,* and *A. brasilense* increased the efficiency of the applied N, managing to reduce N fertilization under proper management practices (Fukami et al., 2018; Galindo et al., 2020; Lee et al., 2020; Blake et al., 2021).

## 5 Conclusion

Topdressing nitrogen fertilization recommended in the crop after succession with grasses is 103 kg ha^−1^ because it provides higher oil yield and grain yield. The recommended topdressing fertilization with nitrogen in the cultivation in succession to legumes without the effect of foliar inoculation is 180 kg ha^−1^ of N. The use of foliar inoculations with *A. brasilense, B. subtilis,* and *P. fluorescens* provided a reduction of 44, 37, and 49% of nitrogen fertilization for grain yield and 49, 40, and 48% of nitrogen fertilization for oil yield in castor bean cultivation in succession to legumes. Foliar inoculation with *B. subtilis* and *A. brasilense* provided the highest grain yield and castor oil yield in cultivation in succession to grasses.

Foliar inoculation with *A. brasilense, B. subtilis,* and *P. fluorescens* provided higher shoot N uptake, grain N uptake, and total N uptake at topdressing rates of 45, 90, and 135 kg ha^−1^ of N in both systems of succession of plants (legumes and grasses).

Despite different aspects of the use of PGPB in a new way in the field condition, our study was limited with a significant progression from *in vitro* conditions to field applications and constraints within the rhizosphere like soil microbiota structure and enzymes. Research with PGPB should focus on understanding the genetic mechanisms regulating growth-promoting processes, genetic modification of plants, chemical genomics strategies, rhizospheric engineering, and colonization with large subpopulations of rhizomicrobiomes may help overcome these constraints. This approach could be essential for evaluating critical microbial molecular components that regulate plant development and facilitate effective PGPB application in the field.

## Data Availability

All the data generated and analyzed during this study are included in this article.

## Footnotes

^1^ https://topbiobrasil.com.br/produtos/micro-bio/
